# Cosmological analogies for geophysical flows, Lagrangians, and new analogue gravity systems

**DOI:** 10.1140/epjc/s10052-023-11292-6

**Published:** 2023-02-10

**Authors:** Valerio Faraoni, Sonia Jose

**Affiliations:** Department of Physics and Astronomy, Bishop’s University, 2600 College Street, Sherbrooke, QC J1M 1Z7 Canada

## Abstract

Formal analogies between the ordinary differential equations describing geophysical flows and Friedmann cosmology are developed. As a result, one obtains Lagrangian and Hamiltonian formulations of these equations, while laboratory experiments aimed at testing geophysical flows are shown to constitute analogue gravity systems for cosmology.

## Introduction

There are unexpected formal analogies between cosmology and geophysical flows. Geophysical flows encompass many natural phenomena, including lava flows, the creep of glacier ice, avalanches, and mud slides. In particular, the analogy between early models of ice caps on a horizontal bed (based on an incorrect ice rheology) [1] and lava domes is well known [2, 3]. These early models of ice caps treated glacier ice as a perfectly plastic material, i.e., a non-Newtonian fluid that does not yield under stress until a certain threshold (“yield stress”) is reached, at which point the material deforms abruptly. This kind of material is nowadays referred to as a Bingham fluid. It is now established that glacier ice instead deforms under stress according to the non-linear Glen law relating the strain rate tensor ${\dot{\epsilon }}_{ij}$ and the deviatoric stresses ${\hat{s}}= ( s_{ij} )$ [4]

1 $$\begin{aligned} {\dot{\epsilon }}_{ij} = {{\mathscr {A}}} \, \sigma _{\textrm{eff}}^{n-1} \, s_{ij} , \end{aligned}$$

where ${{\mathscr {A}}}$ is a constant (that depends on the temperature, crystal orientation, and impurities [5–8]) and

2 $$\begin{aligned} \sigma _{\textrm{eff}} = \sqrt{ \frac{1}{2} \, \text{ Tr } \left( {\hat{s}}^2 \right) } \end{aligned}$$

is the effective stress [5–8]. However, Nye’s discussion applies without change to a Bingham fluid on a horizontal bed, which is a good model for lava flow, and gives the parabolic profile for a lava dome on horizontal bed [2, 3, 9]. (The Bingham fluid is the most common non-Newtonian fluid model to describe lava flows [10–17].) Similarly, the discussion of perfectly plastic glacier ice on a slope, although inadequate to describe an alpine glacier because of the wrong rheology, describes a lava flow on a slope. The corresponding analytical solution of the relevant fluid-mechanical equations appears in the pedagogical literature as a simple example of how different ice rheologies produce different macroscopic glacier profiles [18]. Unbeknownst to the author of [18], this solution is perfectly adequate to describe the flow of lava (a Bingham fluid) on a slope [3]. Below, we elaborate on this analogy.

The ordinary differential equations ruling the longitudinal profiles of glaciers, ice caps, lava flows, or lava domes lend themselves to analogies with the Einstein–Friedmann equations of cosmology. These equations describe the evolution of a spatially homogeneous and isotropic universe in general relativity and constitute the basis of modern (or Friedmann–Lemaître–Robertson–Walker, in short “FLRW”) cosmology. The various solutions describing geophysical flows correspond to different matter contents and curvatures for these universes. Since Lagrangian and Hamiltonians for the equations of FLRW cosmology are known, the cosmic analogy provides a way to identify Lagrangians and Hamiltonians for the differential equations describing the analogous geophysical flows. While analogies between geophysical (and other) systems have been explored in the literature (see [19] for a review), the ones that we examine here are novel.

In the next section we recall the basics of FLRW cosmology for the reader unfamiliar with it. Sect. 3 discusses a Newtonian fluid model of a lava front and the relevant cosmic analogy, Lagrangian, and Hamiltonian. Section 4 studies a Bingham fluid model of a lava front; Sect. 5 discusses Bingham models of lava domes and ice caps on horizontal beds and their cosmic analogues, while Sect. 6 extends the discussion to flows on a slope and Sect. 7 contains the conclusions.

## Basics of FLRW cosmology

We follow the notation of Refs. [20, 21] and use units in which the speed of light is unity. *G* is Newton’s constant and the four-dimensional metric tensor has signature ${-}{+}{+}{+}$.

Under the strong mathematical requirements of spatial homogeneity and isotropy motivated by observations of the cosmic microwave background permeating our universe and by large-scale structure surveys, the four-dimensional geometry of the universe is necessarily given by the FLRW line element, which reads [20–24]

3 $$\begin{aligned} ds^2= & {} g_{\mu \nu } \, dx^{\mu } dx^{\nu }= -dt^2 \nonumber \\{} & {} +a^2(t) \left[ \frac{dr^2}{1-Kr^2} +r^2 \left( d\vartheta ^2 + \sin ^2 \vartheta \, d\varphi ^2 \right) \right] \end{aligned}$$

in comoving polar coordinates $x^{\mu }= \left( t, r, \vartheta , \varphi \right)$, where $g_{\mu \nu }$ is the metric tensor. The scale factor *a*(*t*) describes the expansion history of the universe, while the constant *K* describes the curvature of the 3-dimensional spatial sections (the 3-geometries obtained by setting $dt=0$). If $K>0$ the line element (3) describes closed universes; if $K=0$ it corresponds to Euclidean (flat) spatial sections and, if $K<0$, it describes hyperbolic 3-spaces [20, 21, 23, 24]. All the possible FLRW geometries fall in these three categories classified by the sign of the curvature index *K*.

The cosmological spacetime is curved by its mass-energy content and different matter contents produce different cosmic histories *a*(*t*). The cosmic matter is usually described by a perfect fluid with energy density $\rho (t)$ and isotropic pressure *P*(*t*) related by a barotropic equation of state $P=P(\rho )$. The scale factor *a*(*t*) and the matter variables $\rho (t), P(t)$ satisfy the Einstein–Friedmann equations (i.e., the Einstein equation of general relativity adapted to the symmetric line element (3) [20, 21, 23, 24]

4 $$\begin{aligned}{} & {} H^2 \equiv \left( \frac{{\dot{a}}}{a}\right) ^2 =\frac{8\pi G}{3} \, \rho -\frac{K}{a^2} , \end{aligned}$$

5 $$\begin{aligned}{} & {} \frac{\ddot{a}}{a}= -\, \frac{4\pi G}{3} \left( \rho +3P \right) , \end{aligned}$$

6 $$\begin{aligned}{} & {} {\dot{\rho }}+3H\left( P+\rho \right) =0 , \end{aligned}$$

where an overdot denotes differentiation with respect to the comoving time *t* while $H(t)\equiv {\dot{a}}/a$ is the Hubble function [20, 21, 23, 24]. Given any two of these equations, the third one can be derived from them so that only two equations are independent. For convenience, here we choose the Friedmann Equation (4) and the energy conservation Equation (6) as primary, regarding the acceleration Equation (5) as derived. Therefore, our analogies between geophysical flows and FLRW cosmology will be valid only if the energy conservation Equation (6) is satisfied in addition to the Friedmann Equation (4) (which is easy to verify). This happens when a cosmological fluid satisfying the covariant conservation Equation (6) fills the analogous universe. If this fluid has barotropic equation of state $P=w\rho$ with $w=$ const., the conservation Equation (6) corresponds to an energy density scaling as

7 $$\begin{aligned} \rho (t)=\frac{\rho ^{(0)}}{ \left[ a(t) \right] ^{3(w+1)} } , \end{aligned}$$

where $\rho ^{(0)}$ is a positive integration constant determined by the initial conditions [20–24]. Therefore, to establish the validity of an analogy, it is sufficient to establish the validity of an equation of the form of the Friedmann equation with a fluid source satisfying Eq. (7), or sourced by a mixture of (mutually decoupled) fluids each satisfying Eq. (7).

The Lagrangian and Hamiltonian reproducing the Einstein–Friedmann equation through the Euler–Lagrange or the Hamilton equations are obtained from the action of general relativity with a perfect fluid [20, 21]

8 $$\begin{aligned} S= & {} \int d^4 x \, \sqrt{-g^{(4)}} \left( \frac{R}{16\pi G} +\rho \right) \nonumber \\= & {} 4\pi \int dr\, \frac{r^2}{\sqrt{1-Kr^2}} \,\int dt \, L\left( a, {\dot{a}} \right) \end{aligned}$$

(where $g^{(4)}$ is the determinant of the metric $g_{\mu \nu }$), which yields

9 $$\begin{aligned} L \left( a, {\dot{a}} \right)= & {} \frac{3}{8\pi G} \left( a {\dot{a}}^2 -Ka \right) +a^3 \rho , \end{aligned}$$

10 $$\begin{aligned} {{\mathscr {H}}} \left( a, {\dot{a}} \right)= & {} \frac{3}{8\pi G} \left( a {\dot{a}}^2 + Ka \right) -a^3 \rho , \end{aligned}$$

where the dynamics is constrained and the “scalar” or “Hamiltonian” constraint $\mathcal{H}=0$ must be satisfied [20–22]. We are now ready to build analogies between geophysical flows and FLRW cosmology.

## Newtonian model of a lava front

Lava behaves as a Newtonian fluid near a vent, where it is hotter, but sometimes also the front of a lava flow is modelled as a Newtonian fluid [3]. An analytical Newtonian lava flow model is given in [25] and its analogy with FLRW cosmology was mentioned in Ref. [19], which we report and complete here.

Assume that the lava front is homogeneous and isothermal, that it moves at constant velocity on an inclined plane, and that it extends indefinitely in the transversal (*y*-) direction. Let *x* be a coordinate down-slope, *h*(*x*) be the lava thickness measured along an axis perpendicular to the bed, and *L* be the length of the flow, and assume $h\ll L$ (this shallow fluid approximation is common in glaciology and in the study of geophysical flows). Let $\rho$ and $\eta$ be the lava density and dynamic viscosity coefficient, *g* the acceleration of gravity, $\beta$ the slope of the plane lava bed, and $v_0$ the (constant) velocity of the lava in a reference frame fixed to the ground [25]. When lava is described a Newtonian fluid, the Navier–Stokes equations for laminar flow provide the differential equation for the lava flow profile *h*(*x*) [25]

11 $$\begin{aligned} b_0 \, h^3 h' -a_0 \, h^3 +v_0 \, h =0 \end{aligned}$$

where $$ h' \equiv dh/dx $$ and

12 $$\begin{aligned} a_0= & {} \frac{ \rho g \sin \beta }{3\eta } ,\end{aligned}$$

13 $$\begin{aligned} b_0= & {} \frac{ \rho g \cos \beta }{3\eta } . \end{aligned}$$

The analogy with FLRW cosmology follows from rewriting this equation as

14 $$\begin{aligned} \left( \frac{h'}{h} \right) ^2 = \frac{a_0^2}{b_0^2 \, h^2} + \frac{v_0^2}{b_0^2 \, h^6} -\frac{2a_0 v_0}{b_0^2 \, h^4} , \end{aligned}$$

which is analogous to the Friedmann equation

15 $$\begin{aligned} H^2 = -\frac{K}{a^2} +\frac{8\pi G \rho _\mathrm {(stiff)} }{3a^6} + \frac{8\pi G \rho _\mathrm {(rad)} }{3a^4} \end{aligned}$$

for a universe with negative curvature filled by a stiff fluid with equation of state $P_\mathrm {(stiff)}=\rho _\mathrm {(stiff)}$ and a radiation fluid with $P_\mathrm {(rad)}=\rho _\mathrm {(rad)} /3$ (which has negative energy density, a fact that would be unacceptable in realistic cosmology but is rather immaterial in our formal analogy). The map between lava front and cosmology reads

16 $$\begin{aligned}{} & {} K = - \left( \frac{a_0}{b_0} \right) ^2 = - \tan ^2 \beta , \end{aligned}$$

17 $$\begin{aligned}{} & {} 8\pi G \rho _\mathrm {(stiff)} = \frac{3 v_0^2}{ b_0^2} = 3\left( \frac{3\eta v_0}{\rho g \cos \beta }\right) ^2 , \end{aligned}$$

18 $$\begin{aligned}{} & {} 8\pi G \rho _\mathrm {(rad)} = -\frac{ 6a_0 v_0}{ b_0^2} =- \frac{ 18 v_0 \eta \sin \beta }{\rho g \cos ^2\beta } <0 . \end{aligned}$$

The well-known Lagrangian of the analogous FLRW universe indicates the Lagrangian for the lava flow problem

19 $$\begin{aligned} L \left( h,h'\right) = h \, h'^2 -\frac{2a_0 v_0}{b_0^2 h} +\frac{v_0^2}{b_0^2 h^3} +\left( \frac{a_0}{b_0} \right) ^2 h . \end{aligned}$$

Since *L* does not depend explicitly on *x*, the corresponding Hamiltonian

20 $$\begin{aligned} {{\mathscr {H}}}= h \, h'^2 + \frac{2 a_0 \, v_0}{ b_0^2 h} -\frac{v_0^2}{ b_0^2 h^3} - \left( \frac{ a_0}{ b_0} \right) ^2 h \end{aligned}$$

is conserved. Equation (14) for the lava flow profile is recovered by setting ${{\mathscr {H}}}=0$ (this is the Hamiltonian constraint of the Einstein equations).

An analytic solution of Eq. (11) is [25]

21 $$\begin{aligned} x_0 - x = H_0 \cot \beta \left[ \tanh ^{-1} \left( \frac{h}{H_0} \right) -\frac{h}{H_0} \right] , \end{aligned}$$

where $$ 0 \le x \le x_0 $$ and

22 $$\begin{aligned} H_0 = \sqrt{ \frac{3\eta \, v_0}{\rho g \sin \beta }} = \sqrt{ \frac{v_0}{a_0} } = \sqrt{ \frac{ 2\rho _\mathrm {(stiff)} }{ |\rho _\mathrm {(rad)}|} } . \end{aligned}$$

This equation provides the scale factor *a*(*t*) of the spatially curved analogous universe

23 $$\begin{aligned} \frac{t_0 - t}{\tau } = \tanh ^{-1} \left( \frac{a}{a_*} \right) -\frac{a}{a_*} \end{aligned}$$

for $$ 0\le t \le t_0 $$, where

24 $$\begin{aligned} \tau = \sqrt{ \frac{ 2\rho _\mathrm {(stiff)} }{ |\rho _\mathrm {(rad)} |} }\, \cot \beta = \sqrt{ \frac{2\rho _\mathrm {(stiff)} }{\left| \rho _\mathrm {(rad)} K \right| } } \end{aligned}$$

and $a_* = \sqrt{ 2\rho _\mathrm {(stiff)} / \left| \rho _\mathrm {(rad)} \right| }$. In the limit $v_0\rightarrow 0$, the solution for the (now solidified) lava front degenerates into the trivial straight line $h(x)= \left( x-x_0 \right) \tan \beta$. The analogous FLRW universe is empty and has hyperbolic three-dimensional spatial sections, according to

25 $$\begin{aligned} H^2=-\frac{K}{a^2} , \end{aligned}$$

and linear scale factor $a(t)=\sqrt{|K|} \, t$. This is empty Minkowski spacetime in a hyperbolic foliation (i.e., in accelerated coordinates) in which the three-dimensional space is curved, while the four-dimensional curvature is identically zero [23, 24, 26].

The other limit of the solution (21) for $\beta \rightarrow 0$, in which the bed becomes horizontal, corresponds to zero spatial curvature and the stiff fluid as the only matter source in the cosmic analogy. This limit is interesting because it reproduces the shape of an accretionary wedge in the oceanic crust [27]. Setting $\beta =0$ gives [25] the profile

26 $$\begin{aligned} h(x) \simeq \left[ \frac{9\eta v_0}{\rho g} \left( x_0-x \right) \right] ^{1/3} \end{aligned}$$

for $0\le x \le x_0$. The scale factor of the analogous spatially flat expanding universe reduces to the well-known power-law $a(t) \simeq a_* \left( t-t_0 \right) ^{1/3}$ (with $a_*$ a positive constant) caused by a stiff fluid or a free scalar field [28].

## Bingham fluid model of a lava front

Away from a vent, where lava is cooler and more viscous, it behaves more like a Bingham fluid. Consider now a Bingham model of a lava front flowing down an incline with constant slope $\beta$. Let $\rho , \eta , \sigma _0, g$, and $v_0$ be the lava density, viscosity coefficient, yield stress, the acceleration of gravity, and the speed of the front, respectively. Then the Navier–Stokes equations give [25]

27 $$\begin{aligned} h'= \left( 1- \frac{3H_p}{2h} -\frac{H_N^2}{h^2} \right) \tan \beta , \end{aligned}$$

where

28 $$\begin{aligned} H_p = \frac{\sigma _0}{\rho g \sin \beta } , \quad \quad H_N = \sqrt{ \frac{ 3\eta v_0}{\rho g \sin \beta } } . \end{aligned}$$

Rearranging this equation one obtains

29 $$\begin{aligned} \left( \frac{h'}{h} \right) ^2= & {} \frac{\tan ^2\beta }{h^2} + \left( \frac{9H_p^2}{4} - 2H_N^2 \right) \frac{\tan ^2\beta }{h^4} +\frac{H_N^4 \tan ^2\beta }{h^6} \nonumber \\{} & {} +\frac{3H_p H_N^2\tan ^2\beta }{h^5} -\frac{3H_p\tan ^2\beta }{h^3} . \end{aligned}$$

In the analogous FLRW cosmos, the various terms in the right hand side of Eq. (29) describe, respectively, hyperbolic curvature, radiation with energy density $\rho _\mathrm {(rad)} =\rho _\mathrm {(rad)}^{(0)}/a^4$, a stiff fluid with $\rho _\mathrm {(stiff)} =\rho _\mathrm {(stiff)}^{(0)}/a^6$, a fluid with $w=2/3$, and a dust with zero pressure and $\rho _\mathrm {(dust)}=\rho ^{(0)}_\mathrm {(dust)}/a^3$, where

30 $$\begin{aligned} K= & {} -\tan ^2\beta <0 ,\end{aligned}$$

31 $$\begin{aligned} \frac{8\pi G}{3} \, \rho _\mathrm {(rad)}^{(0)}= & {} \left( \frac{9H_p^2}{4} - 2H_N^2 \right) \tan ^2\beta \nonumber \\= & {} \frac{3\left( 3\sigma _0^2-8\eta v_0 \rho g \sin \beta \right) }{ 4 \rho ^2 g^2 \cos ^2\beta } ,\end{aligned}$$

32 $$\begin{aligned} \frac{8\pi G}{3} \, \rho _\mathrm {(stiff)}^{(0)}= & {} H_N^4 \tan ^2\beta =\left( \frac{3\eta v_0}{\rho g \cos \beta } \right) ^2 ,\end{aligned}$$

33 $$\begin{aligned} \frac{8\pi G}{3} \, \rho _{(2/3)}^{(0)}= & {} 3H_p H_N \tan ^2\beta = \frac{3\sigma _0}{\cos ^2\beta } \, \sqrt{ \frac{3\eta v_0 \sin \beta }{\left( \rho g \right) ^3} } ,\end{aligned}$$

34 $$\begin{aligned} \frac{8\pi G}{3} \, \rho _\mathrm {(dust)}^{(0)}= & {} - 3H_p \tan ^2\beta =- \frac{3\sigma _0 \sin \beta }{\rho g \cos ^2\beta } <0 . \end{aligned}$$

Using the common values for lava $\sigma _0 \simeq 2000$ Pa, $\eta \simeq 10^6$ Pa$\cdot$s, $v_0\simeq 10^{-2}$ m/s, one obtains $3\sigma _0^2 -4\eta v_0 \simeq 1.2 \times 10^7$ Pa, making $\rho _\mathrm {(rad)}^{(0)}$ positive. However the dust fluid always has negative energy density.

## Bingham fluid models of lava domes and ice caps on horizontal beds

A Bingham fluid on a horizontal bed assumes a well-known parabolic profile found by Nye in early studies of ice caps [1]. Nye used the incorrect Bingham (or “perfectly plastic”) rheology for ice, which was later superseded by Glen’s law (1) [4], however the discussion applies without modification to Bingham fluids spreading on a horizontal background, such as a lava dome, and is confirmed by experiments [2, 3].

Consider an axisymmetric flow and let *x* point in the radial direction, while *h*(*x*) is the thickness of the (incompressible) material of density $\rho$ at *x*. The simplified Navier–Stokes equations for stationary state in the thin lava approximation give

35 $$\begin{aligned} \frac{\partial P}{\partial x} = \rho g \, \frac{\partial h}{\partial x} , \end{aligned}$$

where *g* is the acceleration of gravity. The basal stress $\tau _b =-\rho g dh/dx$ is equated to the yield stress $\sigma _0$ everywhere [5–7], giving

36 $$\begin{aligned} \rho g \, \frac{\partial h}{\partial x} = \frac{\sigma _0}{h} , \end{aligned}$$

which has as a solution the parabolic Nye profile [1, 5–7]

37 $$\begin{aligned} h(x)=H\sqrt{ 1-\frac{x}{L} } , \quad \quad H= \sqrt{ \frac{2\sigma _0 \, L}{\rho g} } . \end{aligned}$$

By squaring, Eq. (36) is written as

38 $$\begin{aligned} \left( \frac{h'}{h} \right) ^2 = \left( \frac{\sigma _0}{\rho g} \right) ^2 \, \frac{1}{h^4} , \end{aligned}$$

which is analogous to the Friedmann equation (4) for a spatially flat ($K=0$) FLRW universe filled with blackbody radiation with equation of state $P_\mathrm {(rad)}=\rho _\mathrm {(rad)} /3$ and energy density $\rho _\mathrm {(rad)}(t)=\rho ^{(0)}_\mathrm {(rad)} /a^4$, in the correspondence

39 $$\begin{aligned} h(x) \longleftrightarrow a(t) , \quad \quad \frac{8\pi G}{3} \, \rho ^{(0)}_\mathrm {(rad)} = \left( \frac{\sigma _0}{\rho g} \right) ^2 . \end{aligned}$$

This energy density is positive-definite. In the standard cosmological description, the scale factor is $a(t)=a_0 \sqrt{t-t_0}$ with an increasing function *a*(*t*); for the ice cap model of Nye, the downward slope of the ice corresponds to $h'(x)<0$ and $0\le x \le L$. This profile is reflected about the $x=0$ axis to produce an overall profile symmetric under the reflection $x \rightarrow -x$ and with a cusp at $x=0$, where the left and right derivatives have opposite signs [5–8]. The ice cap model corresponding to the correct Glen law (1) for ice rheology satisfies instead the Vialov equation [5–8, 29]

40 $$\begin{aligned} x \, c(x) = \frac{ 2 {{\mathscr {A}}}}{n+2} \left( \rho g h \left| \frac{dh}{dx} \right| \right) ^n h^2 \end{aligned}$$

where *c*(*x*) describes the accumulation rate of ice per unit of area normal to the vertical direction and per unit time (volume of ice added per unit time and per square meter, i.e., a flux density), $n=3$ for ice creep, and ${{\mathscr {A}}}$ is the constant appearing in the Glen law (1). The Vialov profile is obtained for $c=$ const.$\equiv c_0$,

41 $$\begin{aligned} h(x) = H \left[ 1-\left( \frac{x}{L} \right) ^{ \frac{n+1}{n} } \right] ^{ \frac{n}{2(n+1)} } , \end{aligned}$$

where $$H=h(0)$$, $$h(L)=0$$, and

42 $$\begin{aligned} L= \frac{H^2}{ 2^{ \frac{n}{n+1} } }\left[ \frac{2{{\mathscr {A}}}}{(n+2) c_0 } \, \left( \rho g \right) ^n \right] ^{\frac{1}{n+1} } \end{aligned}$$

(the Lagrangian formulation and cosmic analogy for this equation are presented in [19, 30]).

In the limit $n\rightarrow +\infty$ of plastic ice, the Vialov equation (40) reduces to (38) while the Vialov profile (41) becomes the parabolic Nye profile (37) [5, 29].

## Lava dome on a uniform slope

Consider now the flow of a Bingham fluid over a plane of uniform slope $\beta$, in stationary state and in the shallow lava approximation, building a dome on this slope [9]. The same problem has been approached in glaciology by studying an ice sheet made of plastic ice on a slope [18], although for purely pedagogical purposes since the correct rheology is given by Glen’s law (1) instead of plastic ice. The result of [18] is

43 $$\begin{aligned} hh'-h\sin \beta + h_0=0 \,, \quad \quad h_0= \frac{ \tau _b}{\rho g} . \end{aligned}$$

This equation has the analytical solution (for arbitrary large slope angles $\beta$) [18]

44 $$\begin{aligned} x(h) = L+ \frac{h}{\sin \beta } + \frac{h_0}{\sin ^2 \beta } \, \ln \left( 1-\frac{h}{h_0} \, \sin \beta \right) , \end{aligned}$$

which satisfies the boundary condition $$ h=0$$ at $$x=L$$.

There is a difference between ice caps and lava flows. While precipitation on a glacier is distributed (as described by the function *c*(*x*)), lava erupts from a vent and one must describe both down-slope and up-slope flows from this vent, as is done in theoretical descriptions, which commonly leads to *two* solutions of the relevant differential equation [3]. Although not considered in [18], the up-slope solution can be recovered by changing the sign of the basal stress $\tau _b$, therefore of $h_0$, in Eq. (43). Osmond & Griffiths [9] study a silicic lava dome on a slope (silicic lava has relatively low temperature and high viscosity). The lava thickness *h*(*t*, *x*, *y*) satisfies the equation [3, 9]

45 $$\begin{aligned} \left( \frac{\partial h_1}{\partial x} - \sin \beta \right) ^2 +\left( \frac{\partial h_1}{\partial y} \right) ^2 = \left( \frac{ \sigma _0 }{\rho g h \cos \beta } \right) ^2 \end{aligned}$$

where one assumes symmetry about the $y=0$ line which, by continuity, results in $\partial h_1/\partial y=0$ on the line $x=0$. Here $h_1(x)$ is the vertical position of the lava, not its thickness *h* [3, 9], to which it is related by $h= h_1 \cos \beta$. Solving for the longitudinal lava profile along the $x=0$ line and using the rescaled variables

46 $$\begin{aligned} {\bar{x}}\equiv & {} \left( \frac{ \rho g}{\sigma } \, \sin ^2\beta \cos \beta \right) x , \end{aligned}$$

47 $$\begin{aligned} {\bar{y}}\equiv & {} \left( \frac{ \rho g}{\sigma } \, \sin ^2\beta \cos \beta \right) y , \end{aligned}$$

48 $$\begin{aligned} {\bar{h}}_1\equiv & {} \left( \frac{ \rho g}{\sigma } \, \sin \beta \cos \beta \right) h , \end{aligned}$$

Osmond and Griffiths find the solution [3, 9]

49 $$\begin{aligned} x(h) = \left\{ \begin{array}{ll} {\bar{h}}_1-{\bar{H}}_1 +\ln \left( \frac{1-{\bar{h}}_1}{1-{\bar{H}}_1} \right) &{} \quad \text{ if } \;\; x\ge 0 ,\\ {\bar{h}}_1-{\bar{H}}_1 -\ln \left( \frac{1+{\bar{h}}_1}{1+{\bar{H}}_1} \right) &{} \quad \text{ if } \;\; x\le 0 , \end{array} \right. \end{aligned}$$

which satisfies the boundary condition ${\bar{h}}_1={\bar{H}}_1$ at $x=0$, and where the flow has length ${\bar{L}}_1= -\ln \left| 1-{\bar{H}}_1^2 \right|$ and width ${\bar{W}}_1 \simeq 2\left( 1-\sqrt{ 1-{\bar{H}}_1^2} \right)$ [3, 9]. This solution coincides with the solution (44) of [18] for perfectly plastic ice and was rediscovered in Ref. [31] together with the Nye profile (37).

The cosmic analogy is obtained by rewriting Eq. (44) as

50 $$\begin{aligned} \left( \frac{h'}{h} \right) ^2 = \frac{\sin ^2 \beta }{h^2} + \frac{h_0^2}{h^4} -\frac{2h_0 \sin \beta }{h^3} , \end{aligned}$$

which is analogous to the Friedmann equation

51 $$\begin{aligned} H^2 = -\frac{K}{a^2} + \frac{8\pi G}{3} \left( \frac{ \rho ^{(0)}_\mathrm {(rad)} }{a^4} + \frac{ \rho ^{(0)}_\mathrm {(dust)} }{a^3} \right) \end{aligned}$$

where

52 $$\begin{aligned} K= & {} -\sin ^2 \beta , \end{aligned}$$

53 $$\begin{aligned} \frac{8\pi G }{3} \, \rho ^{(0)}_\mathrm {(rad)}= & {} h_0^2 , \end{aligned}$$

54 $$\begin{aligned} \frac{8\pi G }{3} \, \rho ^{(0)}_\mathrm {(dust)}= & {} -2 h_0\sin \beta <0 . \end{aligned}$$

The effective Lagrangian and‘ Hamiltonian for the lava flow obtained from the analogy are

55 $$\begin{aligned} L= & {} h \, h'^2 + h \sin ^2 \beta +\frac{h_0^2}{h} -2h_0 \sin \beta , \end{aligned}$$

56 $$\begin{aligned} {{\mathscr {H}}}= & {} h \, h'^2 - h \sin ^2 \beta - \frac{h_0^2}{h} +2h_0 \sin \beta , \end{aligned}$$

and Eq. (43) is obtained by imposing the Hamiltonian constraint of general relativity ${{\mathscr {H}}}=0$.

The width of the lava flow in the transverse *y*-direction is obtained [9] for $\partial {\bar{h}}_1 /\partial {\bar{x}}\simeq 0$, which leads to

57 $$\begin{aligned} 1+ \left( \frac{ \partial {\bar{h}}_1}{ \partial {\bar{y}} } \right) ^2 = \frac{1}{ {\bar{h}}_1^2} \end{aligned}$$

and to another cosmic analogy through the analogue of the Friedmann equation

58 $$\begin{aligned} \left( \frac{ {\bar{h}}_1 ' }{ {\bar{h}}_1} \right) ^2 = -\frac{1}{ {\bar{h}}_1^2} +\frac{1}{{\bar{h}}_1^4} \end{aligned}$$

(where now a prime denotes differentiation with respect to ${\bar{y}}$), which describes a spatially closed ($K=+1$) universe sourced by blackbody radiation. The solution of [9]

59 $$\begin{aligned} {\bar{y}} ( {\bar{h}}_1 )= \pm \left( \sqrt{ 1-{\bar{h}}_1^2} - \sqrt{ 1-{\bar{H}}_1^2} \, \right) , \end{aligned}$$

which can be inverted as

60 $$\begin{aligned} {\bar{h}}_1( {\bar{y}} ) = \sqrt{ 1- \left( {\bar{y}} \mp \sqrt{ 1- {\bar{H}}_1^2 }\, \right) ^2 } , \end{aligned}$$

is a classic solution of FLRW cosmology [28] and can be rewritten in the parametric form

61 $$\begin{aligned} {\bar{h}}_1(\eta )= & {} \sin \eta , \end{aligned}$$

62 $$\begin{aligned} {\bar{y}}(\eta )= & {} \pm \sqrt{ 1-{\bar{H}}_1^2} + \cos \eta , \end{aligned}$$

where the parameter $\eta$ corresponds to the conformal time of FLRW cosmology defined by $dt=a d\eta$. This FLRW universe begins at a Big Crunch, reaches a maximum size, and then shrinks and collapses to a Big Crunch, mirroring the transverse profile of the lava dome of finite extension. The corresponding Lagrangian and Hamiltonian are

63 $$\begin{aligned} L_1 \left( {\bar{h}}, {\bar{h}}' \right)= & {} {\bar{h}} \, {\bar{h}}'^2 -{\bar{h}} +\frac{1}{ {\bar{h}} } , \end{aligned}$$

64 $$\begin{aligned} {{\mathscr {H}}}_1 \left( {\bar{h}}, {\bar{h}}' \right)= & {} {\bar{h}} \, {\bar{h}}'^2 +{\bar{h}} - \frac{1}{ {\bar{h}} } . \end{aligned}$$

## Conclusions

The Friedmann equation (4) lends itself to various analogies [19], including the differential equations ruling lava flows, because it resembles the energy conservation equation for a one-dimensional motion. Analogue gravity, in which laboratory scale systems mimic gravitational systems such as black holes, wormholes, and universes that cannot be recreated in the lab, has become a mature area of science (e.g., [64–68]). Analogue gravity systems comprise Bose–Einstein condensates and other condensed matter systems [32–45], fluids [46–58], optical systems [59–63] and even soap bubbles [73, 74] and capillary flow [75]. Specifically, analogues of FLRW cosmology have been found in Bose–Einstein condensates [69–72]. Based on the cosmic analogies presented here, tabletop experiments studying the flow of Bingham fluids of interest in geophysics, which employ slurries descending inclines [3], can constitute analogue gravity systems for cosmology. The most interesting phenomena discovered in analogue gravity thus far involve wave propagation and perturbations, which have led to the discovery of analogue Hawking radiation [55], superradiance [54], and cosmological particle production [35], predicted in quantum field theory in curved spacetime and not directly observable in nature, but other aspects may be disclosed by analogue gravity in the future.

As seen above, negative energy densities for the cosmological fluids analogous to geophysical flows do occur sometimes and they are responsible for the appearance of hyperbolic functions in the scale factor *a*(*t*) (cosmologists are familiar with hyperbolic functions in the presence of a negative cosmological constant or of positive curvature index *K* [20–24]). While these negative densities would be unacceptable for real fluids in Einstein gravity, they can be viable in alternative theories where extra degrees of freedom with respect to general relativity can be described as *effective* fluids not subject to the usual physical requirements imposed on ordinary fluids (e.g., [76–78]). The extension of the cosmological analogies reported here to scalar-tensor and other theories of gravity alternative to general relativity will be pursued elsewhere.

## Data Availability

This manuscript has no associated data or the data will not be deposited. [Authors’ comment: There are no data associated with this work because of its theoretical and mathematical nature.]
